# Endometriosis presenting with recurrent massive hemorrhagic ascites and diagnosed by core needle biopsy: A case report

**DOI:** 10.1097/MD.0000000000015477

**Published:** 2019-05-13

**Authors:** Xue Wang, Yiling Li, Jing Tong, Bing Chang, Yi Zhang, Yanjun Liu, Hao Bing, Liping Guo, Dan Li

**Affiliations:** a Department of Gastroenterology; b Department of Gynaecology and Obstetrics; c Department of Ultrasonography, First Affiliated Hospital of China Medical University, Liaoning Province, China.

**Keywords:** case report, core needle biopsy, endometriosis, moderate anemia, recurrent massive hemorrhagic ascites

## Abstract

**Rationale:**

Recurrent massive hemorrhagic ascites secondary to endometriosis is extremely rare in the medical literature.

**Patient concerns:**

We report the case of a 24-year-old nulliparous woman presenting with severe abdominal distention, massive ascites, moderate anemia, menstrual pain, and an elevated CA-125 level.

**Diagnosis:**

We found a thickened peritoneum in the left lower abdomen by ultrasound during the follow-up period, and endometriosis was subsequently diagnosed by performing core needle biopsy (CNB).

**Interventions and outcomes:**

The patient received medical treatment for endometriosis and had a good response to the treatment.

**Lessons:**

This is the first case in which endometriosis ectopic to peritoneum was diagnosed by CNB. Endometriosis should be considered a differential diagnosis when recurrent massive hemorrhagic ascites occur. CNB should be valued as a method for diagnosing endometriosis.

## 1 Introduction

Endometriosis presenting with recurrent massive hemorrhagic ascites has rarely been reported and is usually diagnosed by pathology through laparoscopy or surgery. These inspection techniques are relatively expensive, risky, and laborious. Needle biopsy is generally used for the diagnosis of endometriosis with lesion masses as clinical symptoms.^[1,2]^ Here, we describe a patient with endometriosis presenting with large hemorrhagic ascites and moderate anemia. Ectopic tissue was found in the peritoneum and diagnosed by core needle biopsy (CNB). This case report aims to raise awareness in gynecologists and gastroenterologists when encountering a similar situation.

## 2 Case report

A 24-year-old nulliparous Nigerian woman presented with rapidly enlarging abdominal distention for 3 months. She also complained of anorexia and fatigue. She had regular menstrual cycles with hypermenorrhea and menstrual pain. There was no significant family history or regular medical history. Physical examination was unremarkable except for the presence of massive ascites. There was no abdominal tenderness, rebound tenderness, or muscle tension. The liver and spleen were normal, and we did not find Douglas nodules during anal palpation.

**Figure 1:**
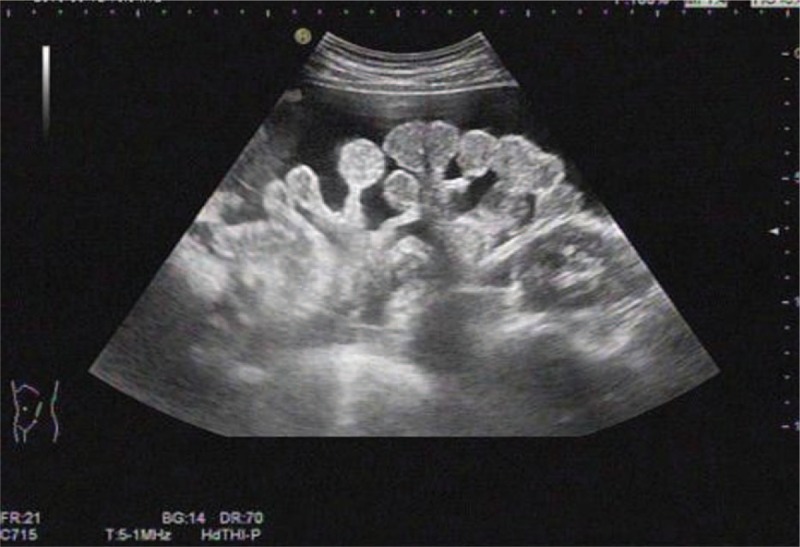
An abdominal ultrasound showing massive ascites.

Blood analysis showed microcytic hypochromic anemia with a hemoglobin of 69 g/dL. Liver function, kidney function, iron level, coagulation, and tumor markers were all unremarkable except for mildly increase in the CA 12-5 level (41.54 U/mL). The human chorionic gonadotropin and purified protein derivative tests were all negative. The platelet count was highly elevated (804 × 10^9^/L), whereas bone marrow smear and culture results had no abnormalities. Two hundred milliliters of dark brown fluid was drained out through paracentesis, and cytological analysis of ascites failed to find any evidence of malignant cells. An ultrasound scan of the abdomen and pelvis revealed massive ascites fluid with no evidence of intestinal and adnexal nodes (Fig. 1). An abdominal contrast computer tomography scan further disclosed extensive ascites. In addition, omental peritoneum ultrasonography, gastrointestinal endoscopy, echocardiography, and ultrasound scan of superficial lymph nodes were all normal. Because of no definite evidence, laparoscopy was recommended, but the patient rejected the examination.

**Figure 2:**
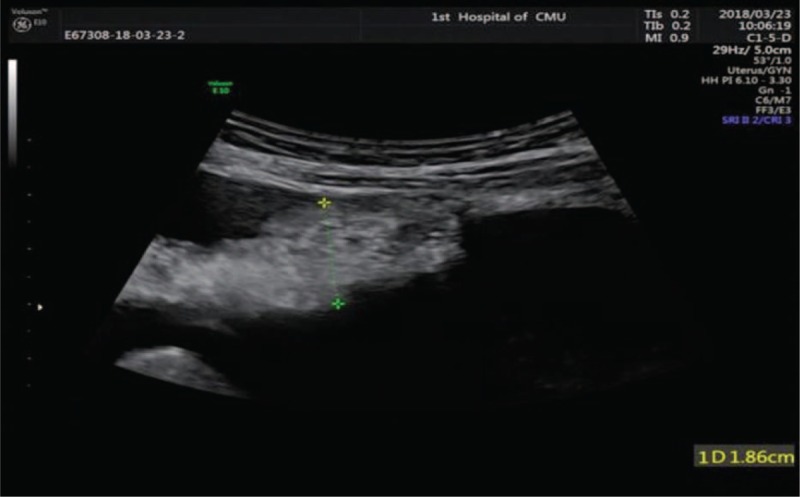
Peritoneal ultrasound shows a thickened uneven echo peritoneum in the left lower abdomen.

**Figure 3:**
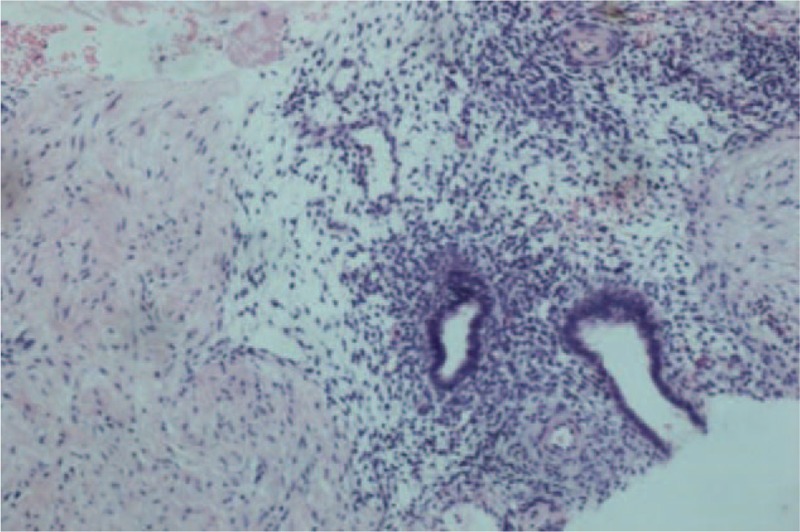
Pathology shows endometrial glandular cells and surrounding stromal cells in the omental tissues [hematoxylin and eosin (HE) staining, 10×].

The patient insisted on a follow-up; the hemorrhagic ascites recurred several times; and the patient required repeated paracentesis to improve symptoms nearly every month. After 1 year, a limited omentum-like echo in the left lower abdomen was found by peritoneal ultrasound. The ultrasound image showed a thickened uneven echo peritoneum of approximately 1.86 cm (Fig. 2). Then, a CNB of the thickened peritoneum was performed with a 14G needle under ultrasound guidance. The removed tissue was sent for pathological analysis. The pathologic diagnosis of endometriosis is based on the presence of 2 or more of the following features: endometrial glandular cells, surrounding stromal cells, and hemosiderin-laden macrophages.^[3]^ In our patient, we observed endometrial glandular cells and surrounding stromal cells in the omental tissues (Fig. 3).

Considering age and fertility requirements, the patient chose conservative medical therapy. She received gonadotropin-releasing hormone (GnRH) analogs leuprorelin acetate for 3 months. The patient has been under contraception treatment of drospirenone and ethinylestradiol tablets for 8 months until now. She also added a polysaccharide iron complex for nearly 2 months. The patient's condition has greatly improved after taking these prescribed medications. Although ascites still exists, the rate of growth has significantly decreased. The hemoglobin level increased to 113 g/dL recently, and the CA-125 level became normal. This therapeutic effect is worth investigating, and we will continually focus on the patient's conditions.

## 3 Discussion

Recurrent hemorrhagic ascites secondary to endometriosis is an extremely rare presentation. The first case of endometriosis-related ascites was described by Brew^[4]^ in 1954; since then, only approximately 60 cases have been reported in the literature. A systematic review and meta-analysis of existing cases reported that 63% of these women had African origins, 82% were nulliparous, and 38.1% presented with pleural effusion. Abdominal distension, anorexia or weight loss, abdominal pain, and menometrorrhagia were the most common symptoms, whereas pelvic mass was the most frequent physical finding.^[5]^

We searched for published articles and found that almost all the endometriosis patients who presented with hemorrhagic ascites were diagnosed by laparoscopy and subsequent pathology. The ectopic sites included broad ligament, intestinal wall, uterus, ovary, peritoneum, and so on. Needle biopsy was usually used for the diagnosis of endometriosis presenting with lesion masses for clinical symptoms. Catalina-Fernández et al introduced 7 patients diagnosed as endometriosis by fine needle biopsy (FNB); 4 patients had a history of gynecological surgery and the lesions located in or near the scar, the remaining patients had lesions that occurred spontaneously in the umbilicus, inguinal, or perineum. All patients were admitted to the hospital with a growing abdominal mass and diagnosed by puncturing the mass.^[1]^ Fulciniti et al reported 10 patients diagnosed with endometriosis by FNB; with the exception of 6 patients who had a solid abdominal mass and the remaining 4 patients had an ovarian cystic mass. In addition, all the puncture sites were visible or radiographic masses.^[2]^ To the best of our knowledge, this case report is the first to puncture the thickened peritoneum and diagnosis endometriosis by CNB. CNB is an accurate, safe, and cost-effective procedure. CNB can be used for excluding or diagnosing malignancy and producing a better preoperative therapeutic plan. Therefore, CNB should be valued as a method for diagnosing endometriosis.

The physiopathology of endometriosis-related ascites is not completely known. Predominant theories include peritoneal irritation from ruptured endometrial implants, subdiaphragmatic lymphatic obstruction, and retrograde menstruation.^[6–8]^ Anemia secondary to this condition is not a usual presentation and may have caused a compensatory increase in platelets in our patient. Furthermore, hemorrhagic shock has been previously reported,^[9,10]^ and reminds us that endometriosis is a life-threatening complication.

The long-term management of recurrent massive ascites caused by endometriosis is difficult. Medical therapy, including GnRH analogs, danazol, and progestogens, has been reported in many cases since 1985.^[11]^ Another treatment option is surgery, which can be performed conservatively or radically. A literature review revealed that endometriosis-related ascites have a high recurrence rate of >50% after unilateral oophorectomy or cystectomy but have no recurrence after bilateral salpingo-oophorectomy.^[12]^ Thus, patients who undergo conservative surgery or medical therapy should be closely followed up. Disease severity, patient age, drug resistance, and personal preference should be considered when choosing medical therapy or surgery approaches. In this case report, the patient is an unmarried and nulliparous college student, and medical therapy is a better option for her to reduce lesions and remove symptoms.

Endometriosis deterioration has been reported with an exacerbation rate of 0.3% to 1%.^[13]^ Endometrioid adenocarcinoma is the most frequently type of cancer outside the ovary, followed by sarcoma and clear-cell tumors.^[14]^ Estrogen stimulation is considered a risk factor. Malignancy should be suspected in patients who have frequent recurrence or rapid development. Therefore, follow-ups are important measures to discover changes in the patient.

In conclusion, endometriosis should be considered as a differential diagnosis when massive hemorrhagic ascites occur in childbearing-aged female patients. Following up with these patients is an important measure to discover the disease progression, and CNB should be valued as a method for diagnosing endometriosis.

## Author contributions

**Conceptualization:** Dan Li.

**Data curation:** Jing Tong, Hao Bing.

**Formal analysis:** Xue Wang, Jing Tong, Liping Guo.

**Investigation:** Yiling Li, Bing Chang, Yi Zhang.

**Methodology:** Bing Chang, Liping Guo.

**Software:** Yanjun Liu.

**Supervision:** Yiling Li.

**Visualization:** Dan Li.

**Writing – original draft:** Xue Wang.

**Writing – review and editing:** Xue Wang.
